# The MdABI5 transcription factor interacts with the *MdNRT1.5*/*MdNPF7.3* promoter to fine-tune nitrate transport from roots to shoots in apple

**DOI:** 10.1038/s41438-021-00667-z

**Published:** 2021-11-01

**Authors:** Ya-Jing Liu, Ning Gao, Qi-Jun Ma, Jiu-Cheng Zhang, Xun Wang, Jing Lu, Yu-Jin Hao, Xiao-Fei Wang, Chun-Xiang You

**Affiliations:** State Key Laboratory of Crop Biology, Shandong Collaborative Innovation Center of Fruit & Vegetable Quality and Efficient Production, College of Horticulture Science and Engineering, Shandong Agricultural University, Tai-An, Shandong 271018 China

**Keywords:** Abiotic, Plant molecular biology

## Abstract

Nitrate is a major nitrogen resource for plant growth and development and acts as both a crucial nutrient and a signaling molecule for plants; hence, understanding nitrate signaling is important for crop production. Abscisic acid (ABA) has been demonstrated to be involved in nitrate signaling, but the underlying mechanism is largely unknown in apple. In this study, we found that exogenous ABA inhibited the transport of nitrate from roots to shoots in apple, and the transcription of the nitrate transporter *MdNRT1.5*/*MdNPF7.3* was noticeably reduced at the transcriptional level by ABA, which inhibited the transport of nitrate from roots to shoots. Then, it was found that the ABA-responsive transcription factor MdABI5 bound directly to the ABRE recognition site of the *MdNRT1.5* promoter and suppressed its expression. Overexpression of *MdABI5* inhibited ABA-mediated transport of nitrate from roots to shoots. Overall, these results demonstrate that MdABI5 regulates the transport of nitrate from roots to shoots partially by mediating the expression of *MdNRT1.5*, illuminating the molecular mechanism by which ABA regulates nitrate transport in apple.

## Introduction

Apple (*Malus domestica*) is an economically important fruit and is widely planted worldwide. Both genetic and environmental factors affect fruit quality and yield, and nutrient absorption and utilization are very important for apple growth. Nitrogen is the most important nutrient promoting crop productivity^1^. In agricultural production, effective utilization of nitrogen is the key to high plant yield, but as a great deal of nitrogen in the soil cannot be absorbed fully by plants, it is necessary to study the mechanisms and genes involved in nitrogen absorption, assimilation, and transport to improve nitrogen use efficiency.

NO_3_^–^ and NH_4 _^+^ are the major nitrogen sources in aerobic and anoxic soils, respectively^2^. For most crops, nitrate is the major nitrogen source^3^, and its uptake and transport by plants have been studied in depth. Nitrate is taken up into plants by nitrate transporters. To adapt to different environmental NO_3_^−^ concentrations in the environment, plants have evolved two NO_3_^−^ absorption systems, low-affinity transport systems, and high-affinity transport systems, with corresponding low affinity (NRT1) and high affinity (NRT2) NO_3_^−^ transporters^4^. In *Arabidopsis*, the NRT1/PTR family contains 53 members, while the NRT2 family contains 7 members^5,6^. Some NRTs have also been functionally identified. For example, NRT1.1/CHL1 is both a nitrate sensor and dual-affinity nitrate transporter^7,8^. NRT1.1, NRT1.2, NRT2.1, and NRT2.2 are involved in nitrate uptake^9–11^, NRT1.4 regulates leaf nitrate homeostasis^11^, NRT1.6 participates in the transfer of nitrate from maternal tissue to developing embryos^12^, NRT1.7 regulates nitrate remobilization from source to sink tissue^13^, and NRT1.9 is involved in the transport of nitrate from roots to shoots^14^.

NRT1.5/NPF7.3 and NRT1.8 have been confirmed as two crucial nitrate transporters for long-distance transport^15^. *NRT1.5* is expressed predominantly in root pericycle cells and plays an important role in loading nitrate into the xylem, while *NRT1.8* is mainly expressed in xylem parenchyma cells and plays a major role in unloading nitrate from xylem sap^10^. Under normal conditions, NRT1.5 loads nitrate from roots into xylem vessels (from which it is transferred to aerial tissues), whereas NRT1.8 unloads nitrate into xylem parenchyma cells^15^. Microarray analysis demonstrated that NRT1.5 and NRT1.8 have opposite regulatory patterns under various abiotic and biotic stresses^4^. Nitrate reallocation to plant roots often occurs under stress; a previous study indicated that drought, cadmium, and salt treatments reduced the expression of *NRT1.5*, and the transcription of the abiotic stress-responsive genes *Na*^*+*^/*H*^*+*^ *EXCHANGER1* (*NHX1*), *SALTS OVERLY SENSITIVE1* (*SOS1*), *RESPONSIVE TO DESICCATION29A* (*RD29A*), and nitrate transporter *NRT1.8* was upregulated in *nrt1.5* mutant plants. These findings indicate that NRT1.5 may be involved in nitrate reallocation to regulate plant stress tolerance^16^.

In plants, nitrate is one of the most abundant anions, and its absorption and transport are believed to be accompanied by K^+^, NRT1.5 plays a crucial role in K^+^ translocation from roots to shoots and is also involved in the coordination of K^+^/ NO_3_^−^ distribution in plants^6,17^. In addition to K^+^/NO_3_^-^, recent research has found that NRT1.5 transports IBA (indole-3-butyric acid) more efficiently than IAA, and IBA is the preferred substrate of NRT1.5 in vivo. NRT1.5-mediated IBA uptake into specific cells is involved in root gravitropism^18^.

Nitrate is both an important nutrient and signaling molecule for regulating the expression of many genes^19^. Nitrate inhibits anthocyanin accumulation primarily by suppressing the expression of *production of anthocyanin pigments* genes which are induced by light in *Arabidopsis*^20^. The bZIP transcription factor *elongated hypocotyl 5* (HY5), which positively regulates plant photomorphogenesis, is involved in nitrogen signaling by promoting the expression of nitrate uptake genes and assimilation-related genes^21^. Nitrate signaling pathways for regulating phosphate utilization have been extensively studied. A previous study found that NRT1.1B could perceive nitrate signals and degrade the repressor protein SPX4, with subsequent release of core transcription factors for phosphate and nitrate signaling to ensure coordinated utilization of N and P in rice^22^. The NIN-like protein NLP7 is a central transcription factor that regulates the expression of NRT1.1 and NRT2.1 in *Arabidopsis*^23^. Ca^2 + ^signaling is also involved in the response to nitrate^24,25^.

Various hormone signals affect plant nitrate contents. For example, the nitrate content of *Arabidopsis* plants changed when they were treated with ethylene and methyl jasmonate^15^. ABA is a crucial hormone that regulates many developmental processes and abiotic stress responses^26^. ABA has been demonstrated to be involved in nitrate signaling, and the expression levels of nitrate-related genes were altered under ABA treatment in *Arabidopsis*^27^. Nitrate provisioning following nitrate starvation increased root-tissue ABA accumulation in wheat, and this enhancement affected the expression of NRT2/NAR genes^28^. These findings demonstrate that there is some correlation between ABA and nitrate.

In this study, we examined the effect of exogenous ABA on the transport of nitrate in apple seedlings and investigated the pertinent regulatory mechanisms. We found that ABA treatment inhibited the transport of nitrate from roots to shoots, and the transcription factor MdABI5 bound directly to the promoter region of the nitrate transporter *MdNRT1.5* and suppressed its expression. In brief, these results indicate that MdABI5 regulates root-to-shoot transport of nitrate by modulating the expression of *MdNRT1.5*, illuminating the molecular mechanism by which ABA regulates nitrate transport in apples.

## Materials and methods

### Plant materials and experimental treatments

“Orin” calli were subcultured on MS medium containing 1.5 mg L^−1^ 6-benzylaminopurine (6-BA) and 0.5 mg L^−1^ 2, 4-dichlorophenoxyacetic acid (2, 4-D) at 25 °C in the dark and subcultured at 18-day intervals. The “Royal Gala” apple cultivar was subcultured monthly at 25 °C, and the MS medium contained 1.5 mg L^−1^ 6-BA, and 0.2 mg L^−1^ naphthylacetic acid. The wild-type *Arabidopsis* plants used were the Columbia (Col-0) type and were grown at 22 °C.

The basic nutrient solution contained 1.0 mM CaCl_2_, 1.0 mM KH_2_PO_4_, 1.0 mM MgSO_4_, 0.1 mM FeSO_4_·7H_2_O, 0.1 mM Na_2_EDTA·2H_2_O, 50 μM MnSO_4_⋅H_2_O, 50 μM H_3_BO_3_, 0.05 μM CuSO_4_⋅5H_2_O, 0.5 μM Na_2_MoO_4_⋅2H_2_O, 15 μM ZnSO_4_⋅7H_2_O, 2.5 μM KI, and 0.05 μM CoCl⋅6H_2_O, supplemented with 5 and 0.1 mM KNO_3_ as the sole N source, and the corresponding concentration of KCl was added to the nutrient solution to maintain the same K concentration (10 mM KCl). For ABA treatment, we exposed apple (*Malus domestica* “Royal Gala”) seedlings to high nitrogen (5 mM KNO_3_) nutrient solutions containing 1.5 mg L^−1^ 6-BA, 0.2 mg L^−1^ naphthylacetic acid, and different concentrations of ABA (0 and 20 μM ABA) for 0, 1, 3, 6, and 12 h, and the gene expression of *MdNRT1.5* was then measured. Apple calli were treated with high nitrogen (5 mM KNO_3_) medium containing 1.5 mg L^−1^ 6-BA, 0.5 mg L^−1^ 2, 4-D, and different concentrations of ABA (0 and 20 μM ABA) for one day, and then the samples were used for the GUS experiment.

The “Royal Gala” apple cultivar was planted in rooting medium (1/2 MS medium containing 0.2 mg L^−1^ IAA, pH = 5.8). After rooting, the seedlings were cultivated in basic nutrient solutions containing 5 mM KNO_3_ for 30 days, and then seedlings of similar size and status were transferred to vermiculite treated with different nitrate concentrations (5 mM and 0.1 mM KNO_3_) with different ABA concentrations (0, 5, and 20 μM ABA) for 50 days under long-day conditions (25 °C, 8/16-h dark/light). The treatment solutions were renewed every 7 days. After 50 days, the shoots and roots were separated, and we washed the roots with ddH_2_O, dried them with filter paper and measured the fresh weights and nitrate content. The shoots and roots were separated and dried at 80 °C for 3 d, and dry weights were measured.

Chinese crabapple (*Malus hupenensis*) seeds were stratified at 4 °C for 30 days. After germination, the seedlings were cultivated in 5 mM KNO_3_ nutrient solutions for 6 weeks, and then seedlings of similar size and status were transferred to vermiculite treated with different nitrate concentrations (5 mM and 0.1 mM KNO_3_) with different ABA concentrations (0, 5, and 20 μM ABA) for 50 days under a long-day condition (25 °C, 8/16-h dark/light). The treatment solutions were renewed every 7 days.

*Arabidopsis* seeds were sown and germinated on MS nutrient medium. After 1 week of growth, seedlings were transferred to different nitrate concentrations (5 mM and 0.1 mM KNO_3_) with different ABA concentrations (0 and 10 μM ABA). These *Arabidopsis* plants were grown under long-day conditions (22 °C, 8/16-h dark/light). The physiological data shown in Fig. S4 were evaluated after 14 days, and the physiological data shown in Fig. 6 were evaluated after 10 days.

### Plasmid construction and plant transformation

The full-length cDNA sequences of MdNRT1.5 and MdABI5 were inserted into the pRI 101-GFP vector and the pCXSN-MYC vector to obtain MdNRT1.5-GFP and MYC-MdABI5 vectors, respectively, for overexpression analysis. These vectors were transformed into *Arabidopsis* and ‘Orin’ calli using *Agrobacterium* (GV4404)-mediated transformation.

To obtain MdABI5- and MdNRT1.5-overexpression apple roots, MdABI5 and MdNRT1.5 were inserted into the vector pK7GWIWG2 with a red fluorescent protein gene and the vector pRI 101-GFP with a green fluorescent protein, respectively. Then these constructs were subsequently transformed into apple seedlings roots with the same growth state as described by Zhao et al.^29^.

### RNA extraction and qRT-PCR

RNA extraction and reverse transcription were carried out as described by Liu et al.^30^. Quantitative real-time polymerase chain reaction (qRT-PCR) was performed using Step One Plus. At least three biological replicates were analyzed for each sample. All primers used are listed in Supplementary Table S1.

### Transcriptome analyses

Apple seedlings were grown on 0.1 mM KNO_3_ (the sole nitrogen source) for 5 days, half of the apple seedlings were treated with 0.1 mM KNO_3_ plus 20 μM ABA for 6 h, and the remaining apple seedlings were left to remain growing on 0.1 mM KNO_3_ medium for 6 h. Total RNA from plants treated with or without ABA was extracted and used for transcriptome analyses. The transcriptome was analyzed with the methods described by Zhang et al.^31^. Log_2_-fold change values >0.58 were considered significant. The significantly repressed (<0.58-fold) and induced (>1.5-fold) genes related to nitrate uptake and transport are shown in Fig. 2b, and the significantly expressed genes (|log2-fold change| > 0.58) are shown in Supplemental Appendix 1.

### GUS-staining assay

To determine whether the transcriptional activity of the *MdNRT1.5* promoter is reduced by ABA and verify that the ABRE core CACGTA sequence is vital for the ABA response, the normal and mutant promoters (ACAAAC in place of CACGTA) of *MdNRT1.5* were cloned into the p1300-GN vector, which contains the GUS reporter gene. The resulting pMdNRT1.5::GUS and pMdNRT1.5::GUS (m) constructs were transiently transformed into apple calli. To verify the activation or suppression of *MdNRT1.5* by MdABI5, 35S::MdABI5 was cotransformed into pMdNRT1.5::GUS calli. GUS staining and activity were used to determine the activity of the transgenic calli as described by Hu et al.^32^.

### ChIP-PCR analyses

35S::MYC and 35S::MYC-MdABI5 transgenic calli were subjected to ChIP experiments. Apple calli were crosslinked with formaldehyde and sonicated to disrupt chromatin. An anti-MYC antibody (Beyotime) was used for Chromatin immunoprecipitation-PCR (ChIP-PCR) as described by Hu et al.^33^. qRT-PCR was performed to detect the enrichment of target DNA fragments. The primers used for ChIP-PCR are listed in Supplementary Table S1.

### Electrophoretic mobility shift assays (EMSAs)

EMSA was conducted as described by Xie et al.^34^. MdABI5 was cloned into the vector pPET32a, The MdABI5-HIS recombinant proteins were expressed in and purified from *Escherichia coli* strain BL21 (DE3). The oligonucleotide probes of the MdNRT promoters were specificity labeled with 3’-biotin as described by Ma et al.^35^. The same unlabeled sequences were used as competitors. The primers used are listed in Supplemental Table S1.

### Yeast one-hybrid (Y1H) assay

Y1H assays were performed as described by Wang et al.^36^. The coding sequence of MdABI5 was inserted into the pGADT7 expression vector, and the promoter fragment of MdNRT1.5 that contained the ABRE-binding motifs was inserted into the pHIS2 reporter vector. Then, constituent vectors were cotransformed into the yeast one-hybrid strain Y187. Individual transformants were screened on Trp-, Leu-, and His-deficient medium (SD-T/-L/-H), and then the positive yeast cells were identified by PCR. The positive yeast strain cells were diluted 10, 100, and 1000 times. Ten microliters of solution with different concentrations were dripped onto a selective medium (SD-T/-L/-H) supplemented with 50 mmol/L 3-aminotriazole (3-AT) for 2–3 days at 28 °C.

### Nitrate content

The nitrate content was measured using the hydrazine reduction method as described by Liu et al.^9^. For nitrate content detection, apple seedlings were treated with nitrogen deficiency treatment for 5 days, and then the apple seedlings were treated with high nitrogen (5 mM KNO_3_) and low nitrogen (0.1 mM KNO_3_) nutrient solutions containing different concentrations of ABA (0, 5, and 20 μM). After a 50-d treatment, the roots and shoots of the apple seedlings were collected. *Arabidopsis* seeds were sown on MS medium. Four days after being sown, seedlings were transferred to different nitrate concentrations (5 and 0.1 mM KNO_3_) with different ABA concentrations (0 and 10 μM) after 10 d of treatment, and then the roots and shoots of *Arabidopsis* were collected. The samples were milled into powder, added to l ml ddH_2_O, and then boiled at 100 °C for 30 min. The samples were centrifuged for 10 min at 13,000 rpm, and then the supernatant was collected to a flow cell. The nitrate content was measured by using an AutoAnalyzer 3 continuous flow analytical system (SEAL Analytical).

### Analysis of root-to-shoot nitrate allocation using ^15^NO_3_^−^

WT and *MdABI5***-**overexpressing apple plants were grown on basic nutrient solutions containing 5 mM KNO_3_ for 30 d and then treated with 20 μM ABA for 12 h. Plant material was washed in 0.1 mM CaSO_4_ for 1 min, labeled for 30 min in 5 mM K^15^NO_3_ or 0.1 mM K^15^NO_3_ medium (99% atom excess of ^15^N; pH 6.0), and washed again in 0.1 mM CaSO_4_ for 1 min. The shoots and roots were separated and dried at 80 °C for 3 d, and ^15^N-NO_3_^−^ contents were analyzed using an isotope-ratio mass spectrometry system (Thermo Scientific, USA)^15^.

### Statistical analysis

The data were obtained from three biological replicates. DPS software was used to analyze the significance of the differences as described by Zhang et al.^37^.

## Results

### Exogenous ABA inhibits the transport of nitrate from roots to shoots

**Fig. 1:**
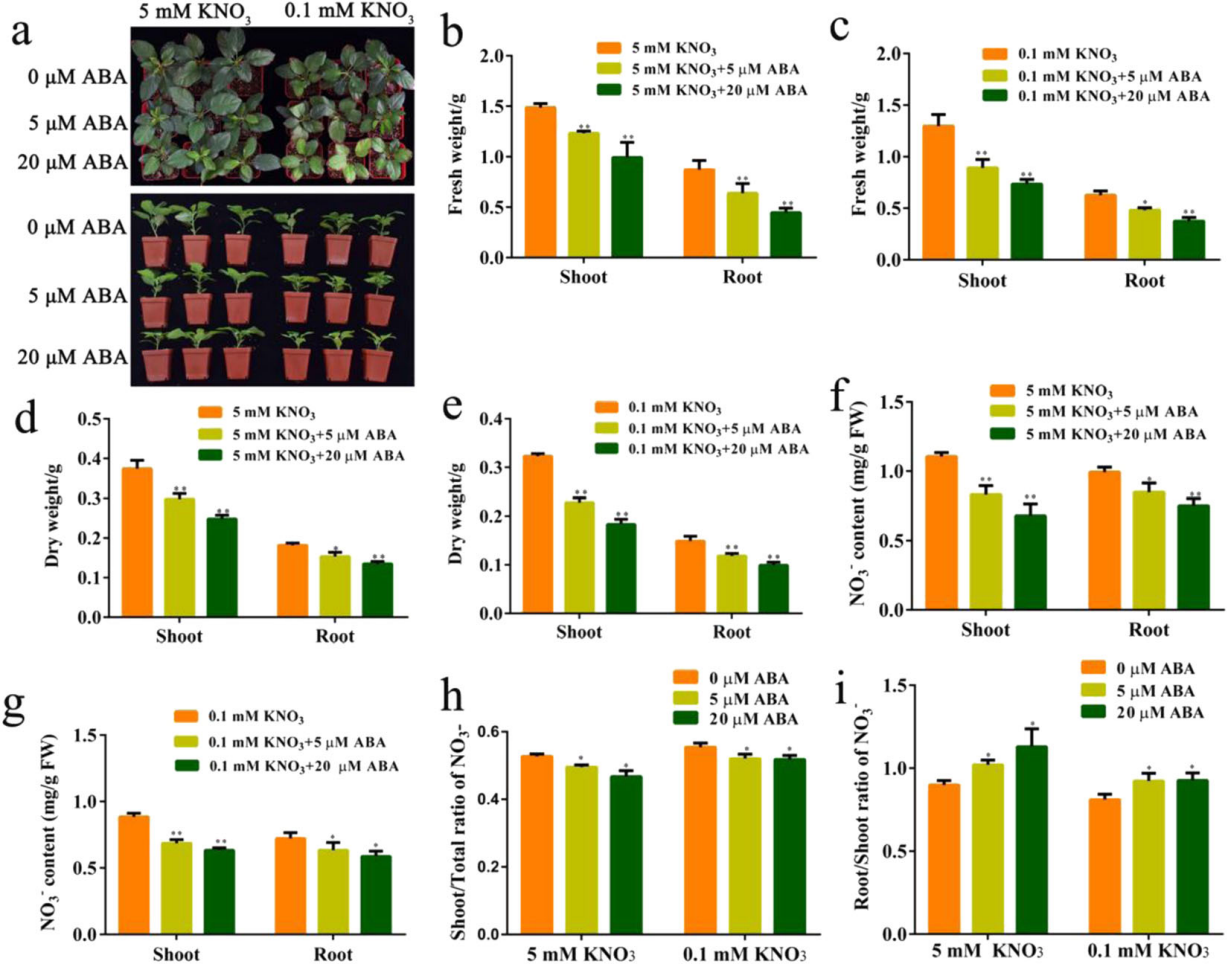
Exogenous ABA inhibits the transport of nitrate from roots to shoots. **a** Phenotype of apple (*Malus domestica* “Royal Gala”) seedlings after growth on high nitrogen (5 mM KNO_3_) and low nitrogen (0.1 mM KNO_3_) nutrient solutions containing different concentrations of ABA (0, 5, and 20 μM) for 50 d. **b**, **c** Fresh weights of shoots and roots after growth on 5 mM KNO_3_ and 0.1 mM KNO_3_ nutrient solutions containing different concentrations of ABA (0, 5, and 20 μM) for 50 d. **d**, **e** Dry weights of shoots and roots after growth on 5 mM KNO_3_ and 0.1 mM KNO_3_ nutrient solutions containing different concentrations of ABA (0, 5, and 20 μM) for 50 d. **f**, **g** Nitrate contents measured in shoots and roots after growth on 5 mM KNO_3_ and 0.1 mM KNO_3_ nutrient solutions containing different concentrations of ABA (0, 5, and 20 μM) for 50 d. **h** Shoot/total ratio of nitrate per apple seedlings. **i** Root/shoot ratio of nitrate per apple seedlings. Data were shown as means ± SE (*n* = 3). Student’s *t* test (*p < 0.05 and **p < 0.01) was used to analyze the statistical significance

To explore the possible effects of ABA on nitrogen uptake and transport, we exposed apple (*M. domestica* “Royal Gala”) seedlings to high nitrogen (5 mM KNO_3_) and low nitrogen (0.1 mM KNO_3_) nutrient solutions containing different concentrations of ABA (0, 5, and 20 μM). After a 50-d treatment, compared with the ABA-free control treatment, shoot growth was lower under ABA treatment (Fig. 1a). After ABA treatment, the fresh weights and dry weights of shoots and roots were clearly decreased (Fig. 1b–e), nitrate contents in both roots and shoots were reduced (Fig. 1f, g), and the shoot/total nitrate ratio was reduced substantially under ABA treatment (Fig. 1h), while the root/shoot nitrate ratio was increased substantially under ABA treatment (Fig. 1i). We performed the same treatments with Chinese crabapple (*M. hupenensis*) seedlings and obtained similar results (Fig. S1). These results indicated that exogenous ABA inhibited the transport of nitrate from roots to shoots in apple seedlings.

### ABA regulates the expression of genes involved in nitrogen uptake and transport

**Fig. 2:**
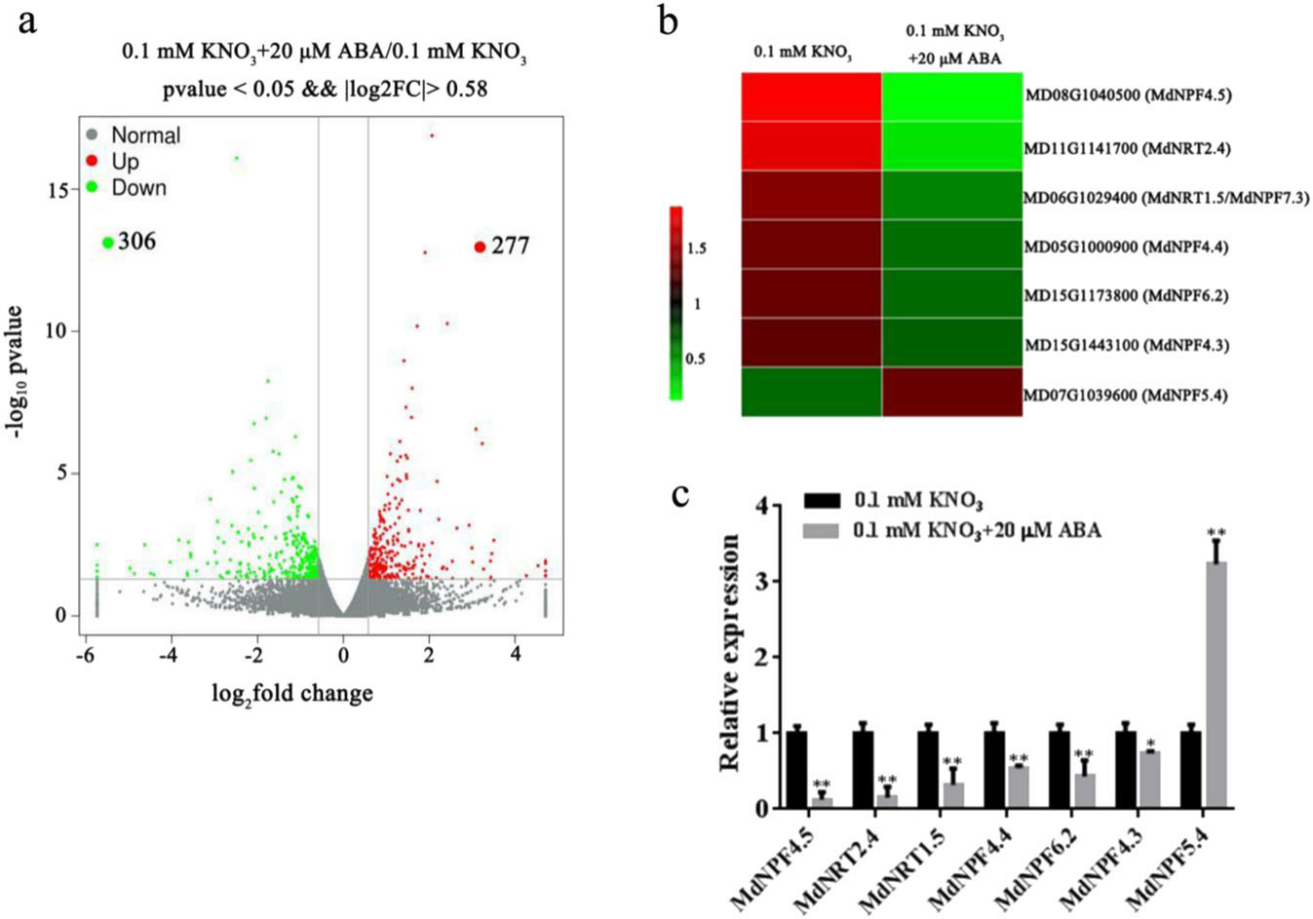
ABA regulates the expression of genes involved in nitrogen uptake and transport. **a** Volcano map indicating up- and downregulation of differentially expressed genes [|Log_2_FC (0.1 mM KNO_3_ + 20 μΜ ABA/0.1 mM KNO_3_) | >0.58; *p* < 0.05] in response to ABA conditions. **b** Transcription levels of nitrate-related genes in seedling roots grown on low nitrate plates (0.1 mM KNO_3_) with or without 20 μM ABA treatment. **c** Quantitative detection of nitrate-related genes expression in the transcriptome

To understand the mechanism of nitrate regulation by ABA, we performed transcriptome analysis with and without ABA. Among the differentially expressed genes we identified [|Log_2_FC| > 0.58; *p* < 0.05], a total of 583 genes demonstrated altered expression (277 up, 306 down) (Fig. 2a; Supplemental Appendix 1); Compared with the levels in untreated controls (without ABA treatment), seven NRT/NPF genes demonstrated altered expression; among these genes, *MdNPF4.5* (GenBank accession number: MD08G1040500), *MdNRT2.4* (MD11G1141700), *MdNRT1.5/MdNPF7.3* (MD06G1029400), *MdNPF6.2* (MD15G1173800), *MdNPF4.4* (MD05G1000900), and *MdNPF4.3* (MD15G1443100) were notably reduced, while *MdNPF5.4* (MD07G1039600) was significantly increased after ABA treatment (Fig. 2b). To further confirm the transcriptome results, we measured the expression of these genes by quantitative PCR (RT-qPCR). Compared with those in the untreated controls (without ABA treatment), the expression levels of *MdNPF4.5*, *MdNRT2.4*, *MdNRT1.5*, *MdNPF6.2*, *MdNPF4.4*, and *MdNPF4.3* were rapidly reduced when apple seedlings were treated with ABA for 6 h (Fig. 2c). These results were consistent with the transcriptome data. *NRT1.5* functions to load nitrate into the xylem to regulate nitrate long-distance transport from roots to shoots^15^. Phylogenetic analysis demonstrated that MdNRT1.5 clustered closely with AtNRT1.5 (Fig. S2a).

Considering that ABA inhibited nitrate transport from roots to shoots and that ABA repressed the transcription of *MdNRT1.5*, MdNRT1.5 was subsequently chosen for further investigation. We fused MdNRT1.5 to the expression vector pRI 101-GFP with a green fluorescent protein and transformed the construct into the roots of apple seedlings in the same growth state using the *Agrobacterium rhizogenes*-mediated transformation method^29^. We examined the fluorescence intensity and expression level of *MdNRT1.5* in overexpression plants and obtained six *MdNRT1.5*-overexpressing apple roots with elevated *MdNRT1.5* transcript levels (Fig. S3a, b). After 30 d of treatment with different concentrations of nitrate (5 mM KNO_3_ and 0.1 mM KNO_3_), no difference was found in the shoot/total nitrate ratio and root/shoot nitrate ratio between transgenic lines and WT under high nitrate (5 mM KNO_3_) treatment (Fig. S3e, f). However, the shoot/total nitrate ratio of the transgenic lines was higher and the root/shoot nitrate ratio was lower under the low nitrate (0.1 mM KNO_3_) treatment (Fig. S3e, f). We performed the same treatments with MdNRT1.5 transgenic *Arabidopsis* seedlings and obtained similar results (Fig. S4). These findings further suggested that the nitrate transporter MdNRT1.5 is able to transport nitrate from roots to shoots.

### ABRE *cis*-elements in the promoter region of the *MdNRT1.5* gene is required for ABA-mediated expression

**Fig. 3:**
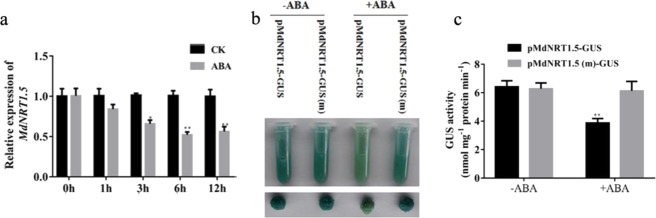
ABRE *cis*-elements in the promoter region of the *MdNRT1.5* gene are required for ABA-mediated expression. **a** MdNRT1.5 transcription levels in apple (“Royal Gala”) treated with 20 μM ABA. **b** GUS staining experiment of pMdNRT1.5-GUS and pMdNRT1.5-GUS (m) transgenic apple calli with or without 20 μM ABA treatment. **c** GUS activity assays of pMdNRT1.5-GUS and pMdNRT1.5-GUS (m) transgenic apple calli

Since the expression of *MdNRT1.5* was inhibited by ABA, to further confirm the transcriptome results, the expression of *MdNRT1.5* in apple seedlings treated with ABA was measured. The results indicated that the expression of *MdNRT1.5* was remarkably reduced after 3 h of ABA treatment (Fig. 3a), further verifying that *MdNRT1.5* expression was inhibited by ABA. We also analyzed the promoter of the *MdNRT1.5* gene, which contains an ABA-responsive element (CACGTA). To explore whether the transcriptional activity of the *MdNRT1.5* promoter was reduced by ABA, the MdNRT1.5 promoter was cloned into the p1300-GN vector, which contains the GUS reporter gene. A pMdNRT1.5::GUS vector was constructed and genetically transformed into apple calli. GUS assays revealed that ABA treatment dramatically reduced the GUS activity, demonstrating that the promoter of *MdNRT1.5* was ABA-responsive (Fig. 3b, c).

To examine whether the ABA-responsive element (ABRE) core CACGTA sequence was vital for the ABA response, a mutated *MdNRT1.5* promoter that contained ACAAAC in place of the wild-type core sequence was artificially constructed and placed in a GUS reporting vector (pMdNRT1.5::GUS (m)) as described previously. We also transformed this mutated construct into apple calli for GUS analysis. The GUS activity of pMdNRT1.5::GUS (m) transgenic calli was unchanged after ABA treatment (Fig. 3b, c), indicating that the ABRE *cis*-element in the *MdNRT1.5* promoter region was critical to the ABA response.

### MdABI5 binds directly to the promoter of *MdNRT1.5* to repress its expression

The ABRE binding factor (ABF)/ABA-responsive element-binding protein (AREB) subfamily belongs to the bZIP transcription factors, which were reported to recognize and bind to ABRE *cis*-elements and regulate ABA-dependent gene expression, AREB proteins play an important role in the ABA response^26^, which motivated us to determine whether AREBs play a role in ABA-mediated regulation of nitrate. The expression of *MdABI5* (LOC103430245), a homolog of AtABI5 (Fig. S2b), was noticeably induced by nitrate (Fig. S5). Therefore, MdABI5 was chosen for further study.

**Fig. 4:**
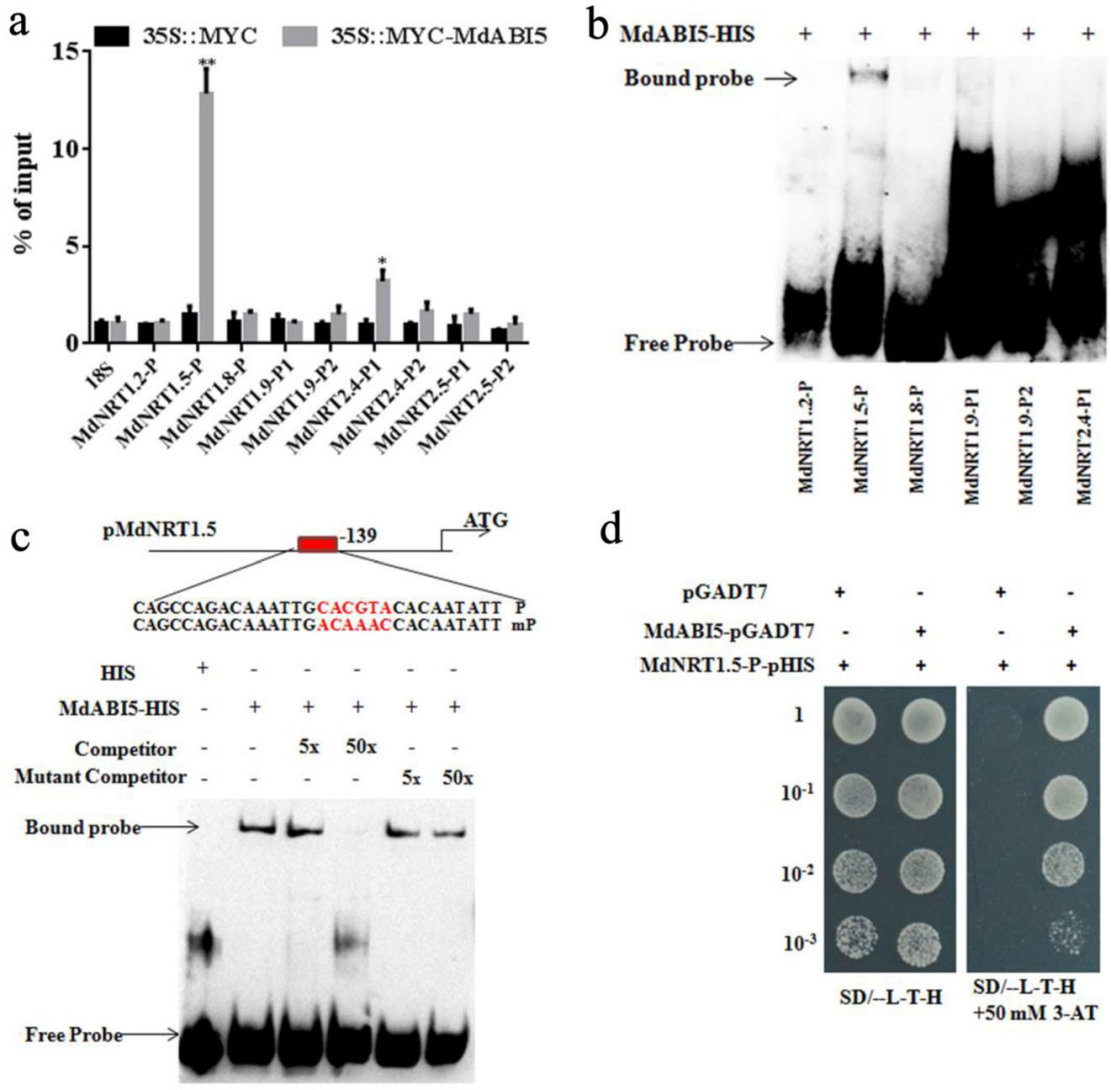
MdABI5 binds directly to the promoter of *MdNRT1.5*. **a** ChIP-PCR assays of the enrichment of nitrate gene promoters in 35S::MYC and 35S::MYC-MdABI5 transgenic apple calli. The 18S served as the loading control. **b** EMSA of the interaction analysis between MdABI5 and labeled DNA probes in the promoters of MdNRT1.2, MdNRT1.5, MdNRT1.8, MdNRT1.9, MdNRT2.4, and MdNRT2.5. **c** The interaction between MdABI5 protein and the *MdNRT1.5* promoter in the EMSA. Mutation probes of pMdNRT1.5 had a mutated ABRE where CACGTA was replaced with ACAAAC. **d** YIH assay of MdABI5 bound to the *MdNRT1.5* promoter in vitro. 10^−^^1^, 10^−^^2^, and 10^−3^ indicate that the yeast concentration was diluted 10, 100, and 1000 times, respectively. 3-AT stands for 3-Amino-1, 2, 4-triazole

To verify whether MdABI5 is bound to the promoters of *MdNRT1.5* and other *MdNRT*s, their promoters were analyzed, and several ABREs were found (Fig. S6a). We subsequently performed ChIP-PCR, which showed that the promoter regions of *MdNRT1.5* and *MdNRT2.4* were enriched in the 35::MYC-MdABI5 transgenic calli compared to the 35S::MYC control (Fig. 4a), indicating that MdABI5 might bind to the ABRE recognition sites of the *MdNRT1.5* and *MdNRT2.4* promoters. To determine whether MdABI5 is bound directly to the *MdNRT1.5* and *MdNRT2.4* promoters, we conducted an EMSA using MdABI5–HIS fusion protein, only an MdNRT1.5 DNA-MdABI5 protein complex was strongly detected (Fig. 4b), and the amount of complex decreased as the amount of the unlabeled ABRE competitor increased. When the ABRE *cis*-element CACGTA was subsequently mutated to ACAAAC, this competition was not observed as the amount of the unlabeled mutant competitor increased (Fig. 4c), indicating specific binding. MdABI5 is also bound directly to the AtNRT1.5 promoter (Fig. S6b).

Yeast one-hybrid analysis was then conducted to further verify the interaction between MdABI5 and the *MdNRT1.5* promoter. Cotransformed yeast cells harboring MdNRT1.5-P-pHIS2 and pGADT7 failed to grow on selective SD medium, but cells harboring MdNRT1.5-P-pHIS2 and MdABI5-pGADT7 grew well on selective medium (Fig. 4d), thereby demonstrating that MdABI5 bound directly to the *MdNRT1.5* promoter.

**Fig. 5:**
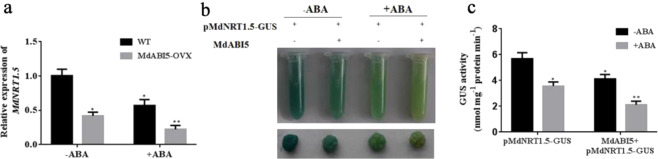
MdABI5 represses the expression of *MdNRT1.5*. **a** *MdNRT1.5* expression levels in *MdABI5*-overexpressing calli with or without ABA treatments. **b** GUS staining experiment of pMdNRT1.5::GUS transgenic calli and transgenic calli containing both pMdNRT1.5::GUS and 35S::MdABI5 with or without ABA treatments. **c** GUS activity assays in transgenic apple calli

To verify the suppression or activation of *MdNRT1.5* by MdABI5, we tested the expression of *MdNRT1.5* in *MdABI5***-**overexpressing calli. The expression of *MdNRT1.5* was decreased in *MdABI5***-**overexpressing calli; in the presence of ABA, the expression of *MdNRT1.5* was decreased further (Fig. 5a). The results indicated that MdABI5 repressed the expression of *MdNRT1.5*. To further verify this result, the 35S::MdABI5 constructs was transiently transformed into the aforementioned pMdNRT1.5::GUS transgenic calli. GUS activity analysis indicated that transgenic calli containing both pMdNRT1.5::GUS and 35S::MdABI5 exhibited much lower GUS activity than calli containing pMdNRT1.5::GUS alone. In the presence of ABA, GUS activity in these transgenic calli was lower, but calli containing both pMdNRT1.5::GUS and 35S::MdABI5 exhibited much lower GUS activity than those containing pMdNRT1.5::GUS alone (Fig. 5b, c). These results demonstrated that MdABI5 repressed the expression of *MdNRT1.5* by binding directly to its promoter region.

### Ectopic expression of *MdABI5* in *Arabidopsis* inhibits ABA-mediated transport of nitrate from roots to shoots

**Fig. 6:**
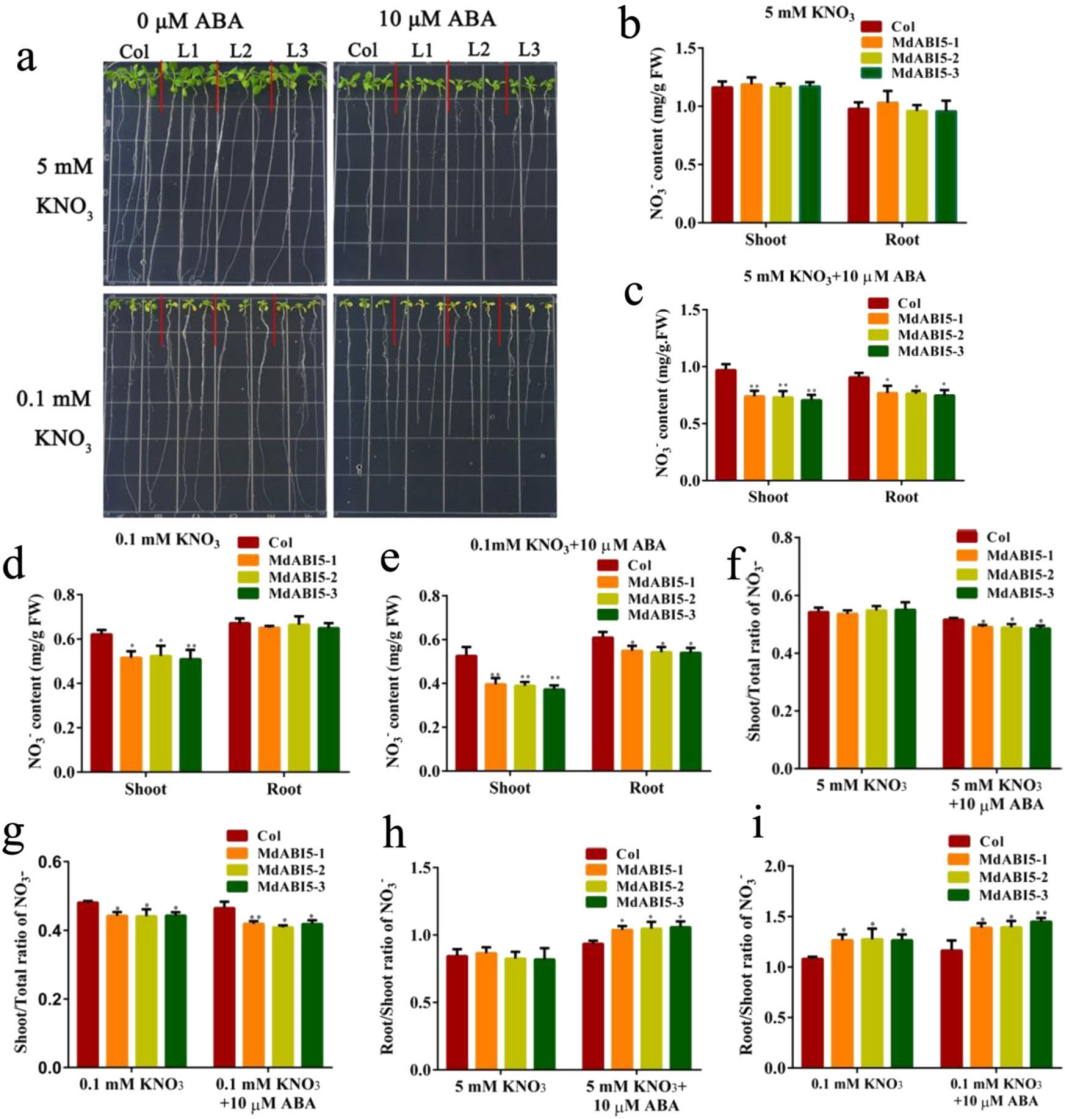
Ectopic expression of *MdABI5* in *Arabidopsis* inhibits the ABA-mediated transport of nitrate from roots to shoots. **a** Phenotype of MdABI5 transgenic lines and Col after growth on high nitrogen (5 mM KNO_3_) and low nitrogen (0.1 mM KNO_3_) nutrient solutions containing different concentrations of ABA (0 and 10 μM) for 10 d. **b**–**e** Nitrate content measured in various plant lines and organs (shoot and root) after growth on 5 mM KNO_3_ and 0.1 mM KNO_3_ nutrient solutions containing different concentrations of ABA (0 and 10 μM) for 10 d. **f**, **g** Shoot/total ratio of nitrate in various plant lines and organs (shoot and root). **h**, **i** Root/shoot ratio of nitrate in various plant lines and organs (shoot and root). Data were shown as means ± SE (*n* = 3). Student’s *t* test (*p < 0.05 and **p < 0.01) was used to analyze the statistical significance

To identify whether MdABI5 was involved in the transport of nitrate from roots to shoots, 35S::MdABI5 was constructed and genetically transformed into *Arabidopsis*, and three independent *MdABI5* transgenic lines with elevated transcription were obtained (Fig. S7a). *MdABI5* transgenic *Arabidopsis* lines showed much lower levels of *AtNRT1.5* transcription than untransformed controls (Fig. S7b). After ABA treatment, the transgenic lines’ fresh weight, root length, and nitrate content were lower than those of controls (Fig. 6 b-e, S8), their shoot/total nitrate ratio was reduced and root/shoot nitrate ratio was increased significantly under both low and high nitrogen conditions (Fig. 6f-i). These results indicated that MdABI5 inhibited ABA-mediated transport of nitrate from roots to shoots.

### MdABI5 enhances ABA-mediated repression of root-to-shoot nitrate transport

**Fig. 7:**
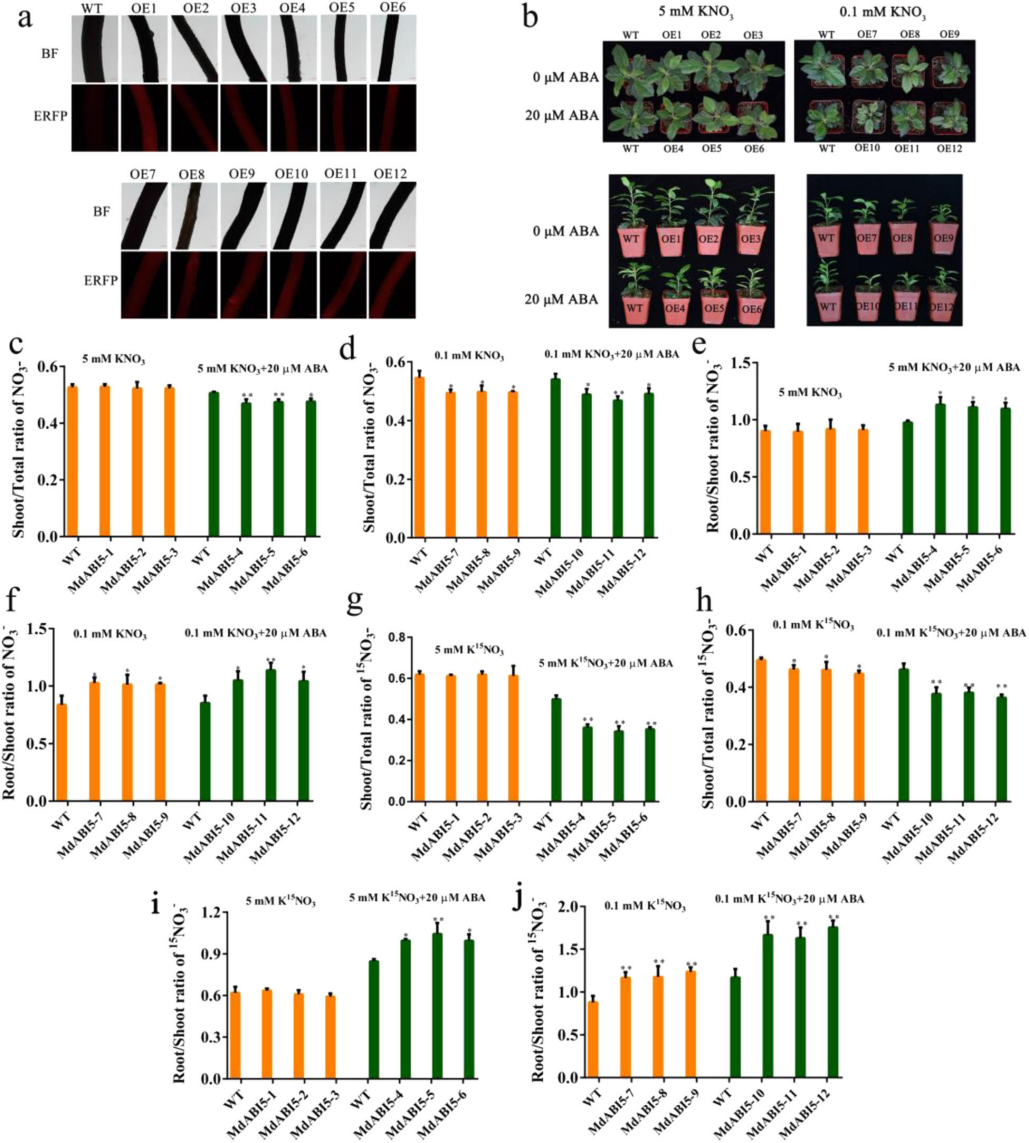
MdABI5 enhances ABA-mediated repression of root-to-shoot nitrate transport. **a** RFP-red fluorescence intensity of apple roots. WT represents ‘Gala’ plants transformed with an empty vector containing an RFP tag; OE represents *MdABI5*-overexpressing apple roots. 1–12 represent different plant lines. **b** Phenotype of WT and *MdABI5*-overexpressing plants after growth on high nitrogen (5 mM KNO_3_) and low nitrogen (0.1 mM KNO_3_) nutrient solutions containing different concentrations of ABA (0 and 20 μM) for 50 d. **c**, **d** Shoot/total ratio of nitrate in various plant lines (WT and *MdABI5*-overexpressing plants) after growth on 5 mM KNO_3_ and 0.1 mM KNO_3_ nutrient solutions containing different concentrations of ABA (0 and 20 μM) for 50 d. **e**, **f** Root/shoot ratio of nitrate in various plant lines (WT and *MdABI5*-overexpressing plants). **g**, **h** WT and *MdABI5*-overexpressing plants were treated with 0 and 20 μM ABA for 12 h and then labeled with different concentrations of K^15^NO_3_ (5 mM and 0.1 mM) for 30 min. The shoot/total ratio of nitrate (^15^NO_3_^−^) was calculated. **i**, **j** The root/shoot ratio of nitrate (^15^NO_3_^−^) was calculated

To test whether MdABI5 was involved in the response of ABA-repressed transport of nitrate in apple, we fused *MdABI5* to the expression vector pK7GWIWG2 with an ERFP in its C-terminal region and transformed the construct into the roots of apple seedlings with the same growing state using the *Agrobacterium rhizogenes*-mediated transformation method^29^. We examined the fluorescence intensity and expression level of *MdABI5* in overexpressed plants and obtained twelve *MdABI5*-overexpressing apple roots with elevated *MdABI5* transcript levels (Figs. 7a and S7c). We then grew both WT and *MdABI5*-overexpressing plants in high nitrogen (5 mM KNO_3_) and low nitrogen (0.1 mM KNO_3_) nutrient solutions containing different concentrations of ABA (0 and 20 μM). After a 50-d treatment, the nitrate content, shoot/total nitrate ratio, and root/shoot nitrate ratio were measured (Figs. 7c–f and S9), and there was no difference in nitrate content, shoot/total nitrate ratio, or root/shoot nitrate ratio between WT and *MdABI5*-overexpressing plants under high nitrogen conditions (Figs. 7c, e and S9a). However, under low nitrogen conditions, the *MdABI5*-overexpressing plants had lower nitrate contents, lower shoot/total nitrate ratios, and higher root/shoot nitrate ratios than did the WT plants (Figs. 7d, f and S9c). Compared with the ABA-free treatment, ABA treatment reduced the nitrate content and shoot/total nitrate ratio and significantly increased the root/shoot nitrate ratio (Figs. 7c–f and S9). To further prove the function of MdABI5, WT, and *MdABI5*-overexpressing plants were treated with 0 and 20 μM ABA for 12 h and then labeled with different concentrations of K^15^NO_3_ (5 and 0.1 mM) for 30 min. Then the shoot/total nitrate (^15^NO_3_^−^) ratio and root/shoot nitrate (^15^NO_3_^−^) ratio were measured and similar results were obtained (Fig. 7g–j). These results indicated that MdABI5 enhanced ABA-mediated repression of nitrate transport from roots to shoots.

## Discussion

Phytohormone signaling molecules are involved in nitrate responses. Recent evidence has provided information concerning how the integration of nitrogen and phytohormone signals gives rise to changes in plant morphology and physiology. Among the various signals that regulate the expression of NRT genes, it has been proposed that auxins, cytokinins (CK), and ABA coordinate nitrogen acquisition^12,38^. For instance, AtNRT2.1 is induced by sugars and nitrate and repressed by CKs^39^. The evidence consistently indicates that CK is a root-to-shoot long-distance signal that represses transcription of *AtNRT1.5*, hence, it may be inferred that inhibition of *AtNRT* by CK reduces nitrate uptake activity^27^. Auxin may also mediate nitrogen uptake and transport because the expression of many NRT genes changes under IAA treatment^40^. In addition, the biosynthesis of CK and the biosynthesis and transport of auxin are affected by nitrate^27^. In addition, NRT1.1/CHL1 also promotes cell-to-cell auxin transport, providing another link between nitrate and auxin^41^.

There is some important evidence linking nitrogen and ABA in some plant species. Several findings provide genetic evidence that ABA is involved in lateral root development under high nitrate treatment in *Arabidopsis*^27^. Previous studies have shown that high-nitrate inhibition is significantly lower in ABA-deficient and ABA-insensitive mutants^42^. A previous study provided additional evidence for a link between nitrogen and ABA signaling in *Medicago truncatula*. A *latd* mutant (the LATD gene belongs to the NRT family and encodes a transporter) showed severe defects in root growth and root meristem maintenance that was rescued by providing exogenous ABA^43^. Another previous study demonstrated that knockdown of *TabZIP60* (which is most closely related to the ABRE-binding factors AtABF2/AtAREB1, AtABF3, and AtABF4/AtAREB2) increased nitrogen uptake and grain yield, however, overexpression of *TabZIP60* had the opposite effects^44^. Our study showed that treatment with exogenous ABA inhibited nitrate transport from roots to shoots in apple (Fig. 1) and reduced the transcription of multiple nitrate-related genes (Fig. 2b, c), providing evidence for a link between nitrate and ABA signaling.

In plants, K^+^/NO_3_^−^ absorption, and transport are somehow coordinated and NRT1.5 is capable of transporting nitrate and potassium^6^. In addition, NRT1.5/NPF7.3 is an IBA transporter involved in root gravitropism^18^. Nitrate is both a crucial nutrient and a signaling molecule for plants. Many molecular components have been identified that are involved in nitrate signaling, including NLP6, NLP7, the TGA transcription factors TGA1 and TGA4, and squamosa promoter binding protein-like9 (SPL9)^25,45,46^. However, nitrate signaling is not clearly understood in apple and other fruit crops. *MdABI5* expression was strongly induced after exposure to nitrate (Fig. S5). In addition to MdABI5, TaWabi5 in wheat (recognized as a putative ortholog of AtABI5) was also found to be nitrate inducible^28^, and ABI5 in *Arabidopsis* is involved in regulating C/N cross-talk and controlling lateral root development in response to nitrate^44^. In our study, phylogenetic analysis revealed that MdABI5 is closely related to MdAREB1 and MdAREB2 (Fig. S2b), again suggesting the importance of ABREs in nitrate signaling.

Nitrate reallocation to plant roots often occurs under stress conditions. Under stress, NRT1.5 expression is decreased in roots, inhibiting the transport of nitrate from roots to shoots and allowing nitrate accumulation in roots. This stress-initiated nitrate allocation to roots enhances plant stress tolerance^47^. Functional disruption of NRT1.5 enhances resistance to abiotic stresses; hence, these data indicate that stress-initiated downregulation of NRT1.5 leads to retention of nitrate in roots and improves plant stress tolerance^16^. Because exogenous or endogenous ABA generally stimulates jasmonic acid (JA) biosynthesis, it is believed that JA and ABA work synergistically in various biological processes^48^. JA downregulates the expression of *NRT1.5* and inhibits nitrate transport from roots to shoots^15^. In our study, we found that (i) ABA-induced *MdABI5* expression, (ii) MdABI5 inhibited the expression of *MdNRT1.5* and regulated nitrate distribution in roots under ABA stress, and (iii) *MdABI5* overexpression lines maintained a higher root nitrate proportion than WT when plants were treated with ABA. ABA-initiated nitrate allocation to roots would enable plants to respond and adapt to stress, enhancing plant stress tolerance. Thus, our data support the hypothesis that nitrate reallocation to roots is regulated by NRT1.5 genes and maybe a common stress response. It is possible that ABA and JA synergistically regulate nitrate reallocation, but we did not examine this possibility here.

After treatment with ABA, the expression levels of *MdNRT2.4* and *MdNRT1.5* were rapidly reduced (Fig. 2b, c), consistent with previous results^15,27^. Léran et al. found that the phosphatase ABI2 positively regulated NRT1.1/NPF6.3. In that study, ABA potentially reduced nitrate uptake by inactivating ABI2, which could be crucial for plant survival under stress conditions such as osmotic and drought stress, since nitrate absorption and utilization are energy-intensive processes^49^. These aforementioned studies demonstrate that ABA potentially represses nitrate uptake. In our study, we did not find direct binding of MdABI5 to the promoter of *MdNRT2.4* in apple, whereas the expression of *MdNPF4.5* was significantly decreased after ABA treatment (Fig. 2b, c). As OsNPF4.5 was found to promote the absorption of nitrate in rice^50^, we speculate that MdABI5 may regulate nitrate absorption by binding to the promoter of *MdNPF4.5*, but verifying this requires further study. Future work in this direction will not only improve our understanding of nitrate signaling but will also provide connections between the ABA and NO_3_^−^ signaling pathways.

Overall, in this study, we found that MdABI5 was ABA-responsive and bound directly to the ABRE recognition site of the *MdNRT1.5* promoter, repressing its expression and promoting ABA-mediated inhibition of nitrate transport from root to shoot. Our findings provide new insight into the molecular mechanism by which ABA regulates nitrate transport in apple.

## Supplementary information

Supplementary data

Supplemental Appendix 1
