# Supplementary material for: The MdABI5 transcription factor interacts with the MdNRT1.5/MdNPF7.3 promoter to fine-tune nitrate transport from roots to shoots in apple
Source: Hortic Res. 2021 Nov 1;8:236. doi: 10.1038/s41438-021-00667-z (PMC8558332; doi:10.1038/s41438-021-00667-z)
Supplement: Supplementary file 1 — Supplementary data [file 41438_2021_667_MOESM1_ESM.docx]

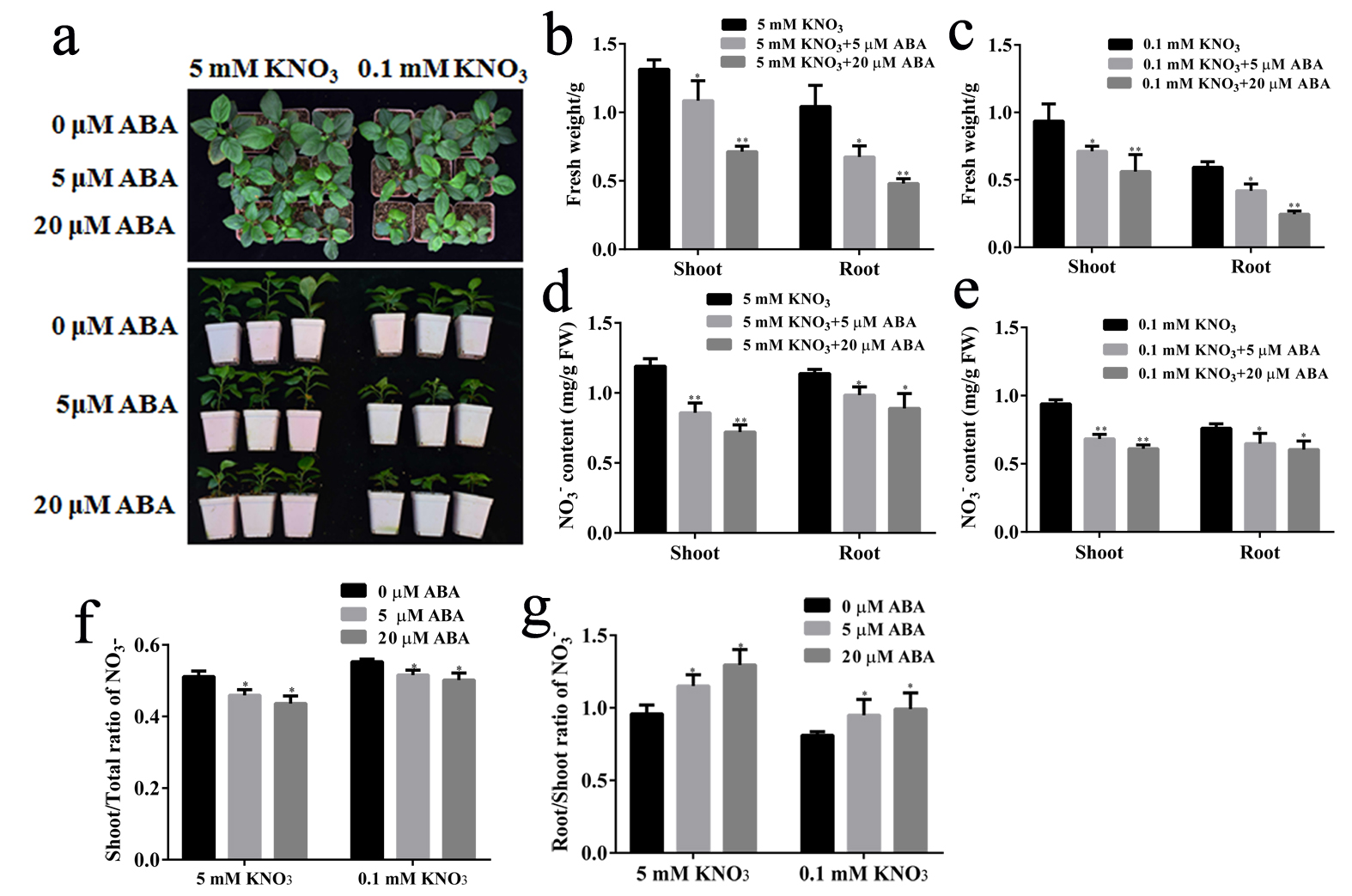


**Fig. S1 Exogenous ABA inhibits the transport of nitrate from roots to shoots**

**a** Phenotype of apple (*Malus hupenensis*) seedlings after growth on high nitrogen (5 mM KNO_3_) and low nitrogen (0.1 mM KNO_3_) nutrient solutions containing different concentrations of ABA (0 μM, 5 μM, and 20 μM) for 50 d. **b-c** Fresh weights of shoots and roots after growth on 5 mM KNO_3_ and 0.1 mM KNO_3_ nutrient solutions containing different concentrations of ABA (0 μM, 5 μM, and 20 μM) for 50 d. **d-e** Nitrate contents measured in shoots and roots after growth on 5 mM KNO_3_ and 0.1 mM KNO_3_ nutrient solutions containing different concentrations of ABA (0 μM, 5 μM, and 20 μM) for 50 d. **f** Shoot /total ratio of nitrate per apple seedlings. **g** Root/shoot ratio of nitrate content per apple seedlings. Data were shown as means ± SE (n =3). Student’s t test (*P < 0.05 and**P < 0.01) was used to analyze statistical significance.


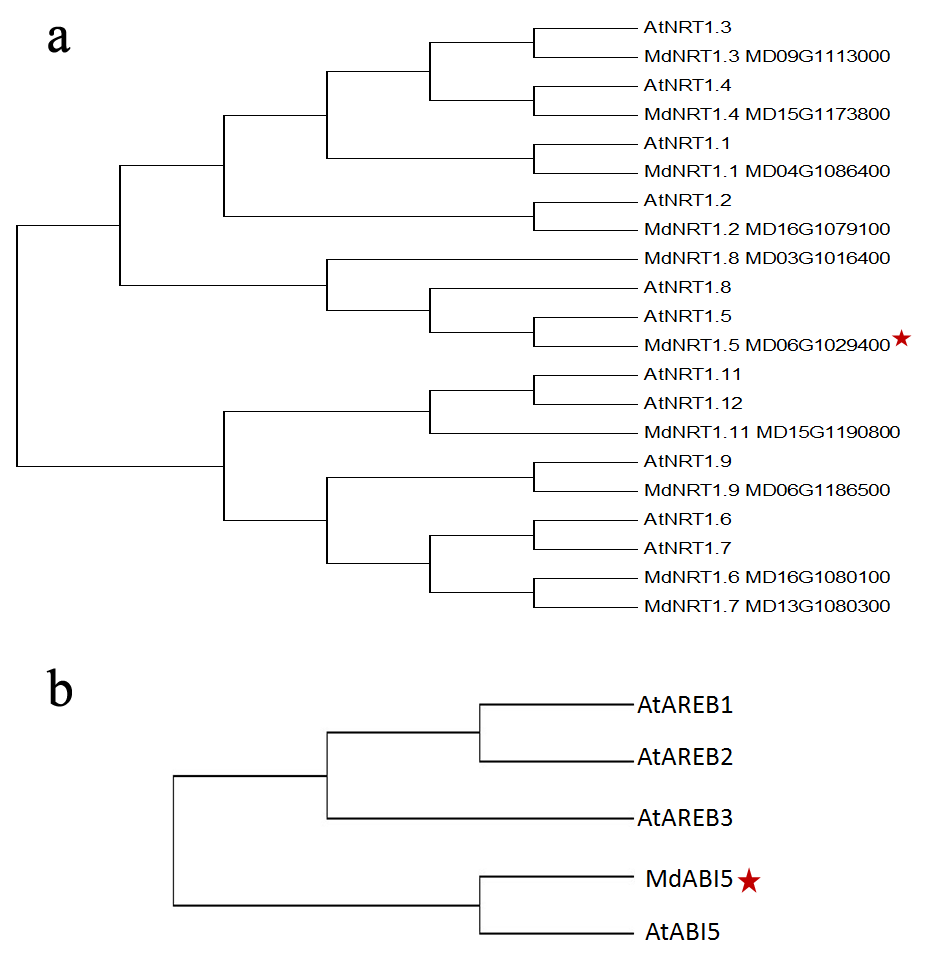


**Fig. S2 Phylogenetic tree analysis. a** Phylogenetic tree of nitrate transporters MdNRTs and AtNRTS. A BLAST search against the apple genome database (The Apple Gene Function & Gene Family DataBase v1.0) was performed. *Arabidopsis* NRTs were used as query, respectively. **b** Phylogenetic tree between apple MdABI5 and *Arabidopsis* AREB proteins. The tree was produced using MEGA5.0 with the amino acid.


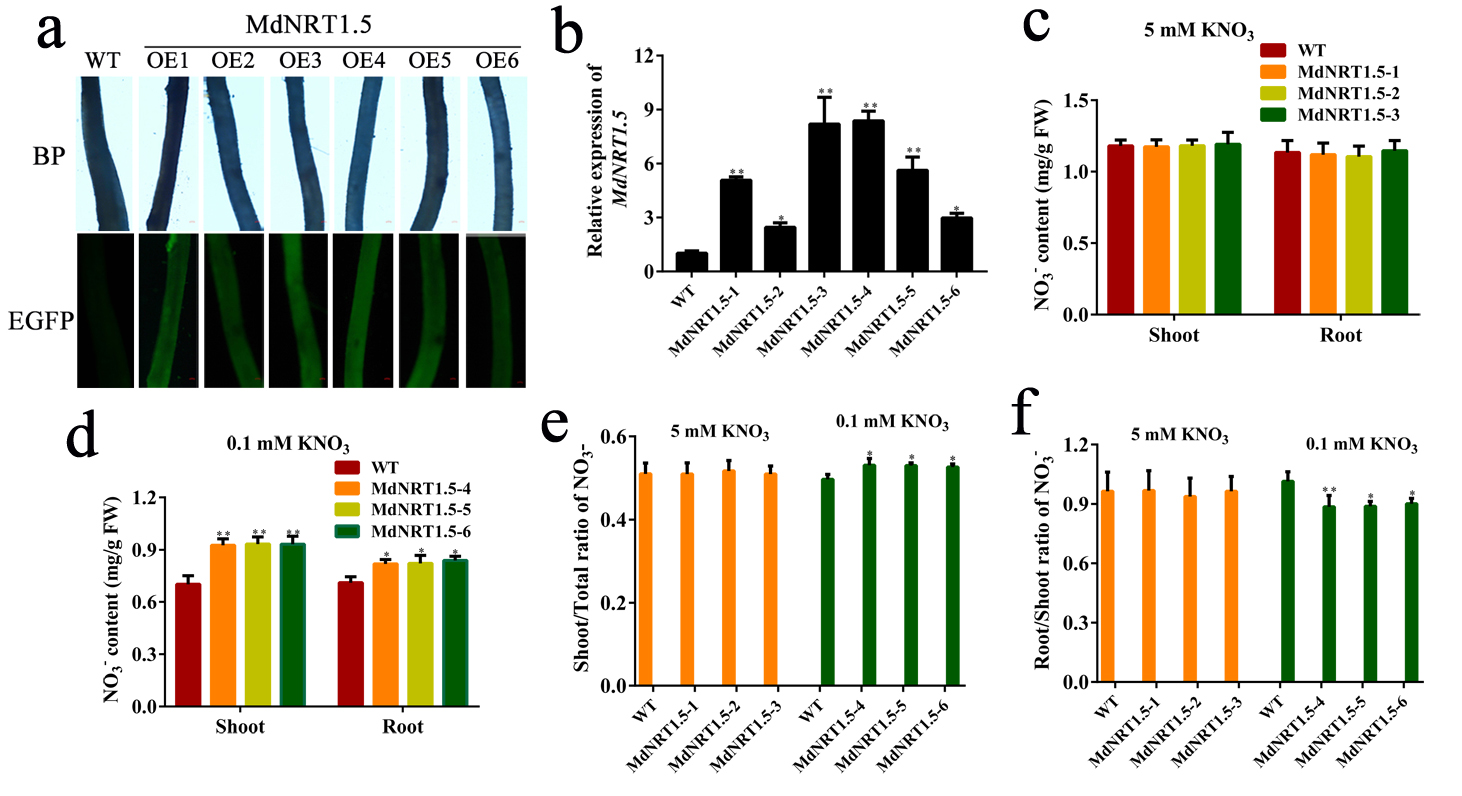


**Fig. S3 MdNRT1.5 promotes the transport of nitrate from roots to shoots. a** GFP-green fluorescence intensity of apple roots. WT represents ‘Gala’ plants transformed with an empty vector containing a GFP tag; OE represents *MdNRT1.5*-overexpressing apple roots. 1-6 represent different plant lines. **b** qRT-PCR determination of the *MdNRT1.5* expression level in *MdNRT1.5*-overexpressing apple roots**. c-d** Nitrate contents measured in various plant lines and organs (shoot and root) after growth on high nitrogen (5 mM KNO_3_) and low nitrogen (0.1 mM KNO_3_) nutrient solutions for 30 d. **e** Shoot/total ratio of nitrate in various plant lines and organs (shoot and root). **f** Root/shoot ratio of nitrate in various plant lines and organs (shoot and root).


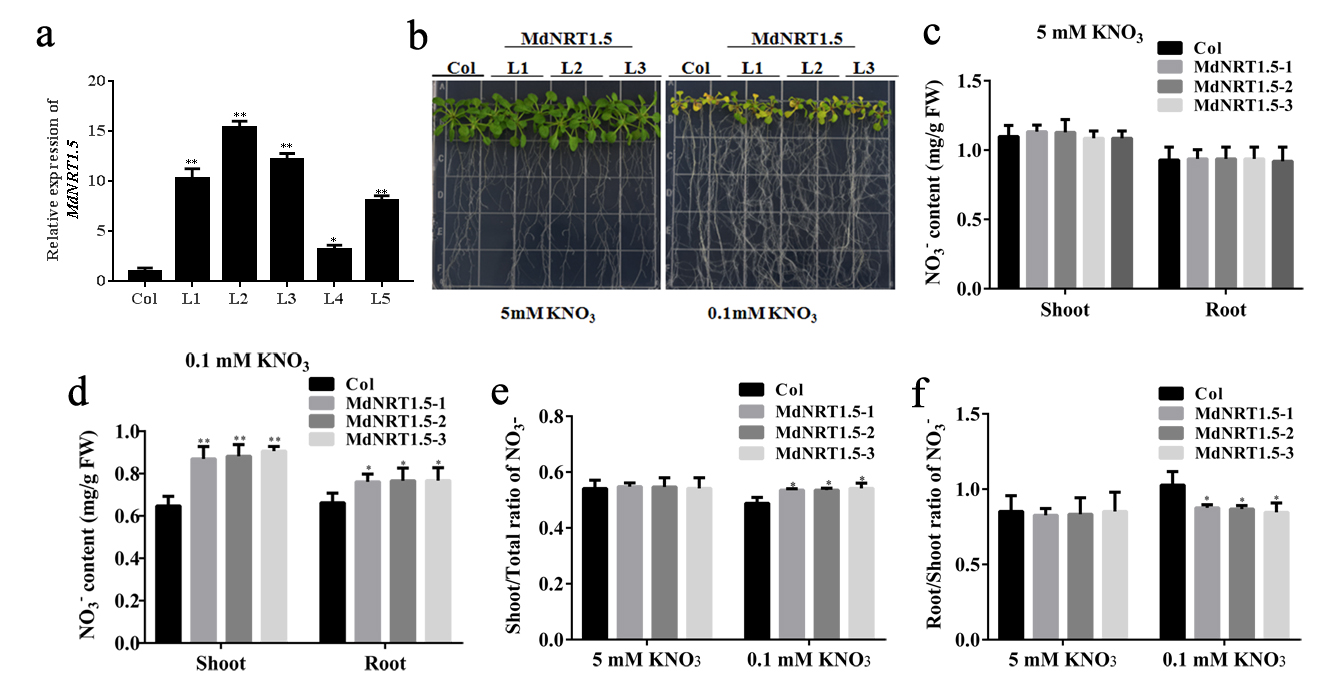


**Fig. S4 MdNRT1.5 transgenic Arabidopsis promotes the transport of nitrate from roots to shoots. a** Determination of *MdNRT1.5* expression level in transgenic *Arabidopsis*. Col represents wild-type Arabidopsis, and L1-L5 represents *MdNRT1.5*-overexpressing *Arabidopsis*. **b** Phenotype of Col and *MdNRT1.5*-transgenic *Arabidopsis* after growth on high nitrogen (5 mM KNO_3_) and low nitrogen (0.1 mM KNO_3_) medium for 14 d. **c-d** Nitrate contents measured in various plant lines (Col and *MdNRT1.5*-transgenic *Arabidopsis*) and organs (shoot and root) after growth on 5 mM KNO_3_ and 0.1 mM KNO_3_ medium for 14 d. **e** Shoot/total ratio of nitrate in various plant lines. **f** Root/shoot ratio of nitrate in various plant lines. Data were shown as means ± SE (n =3). Student’s t test (*P < 0.05 and**P < 0.01) was used to analyze statistical significance.


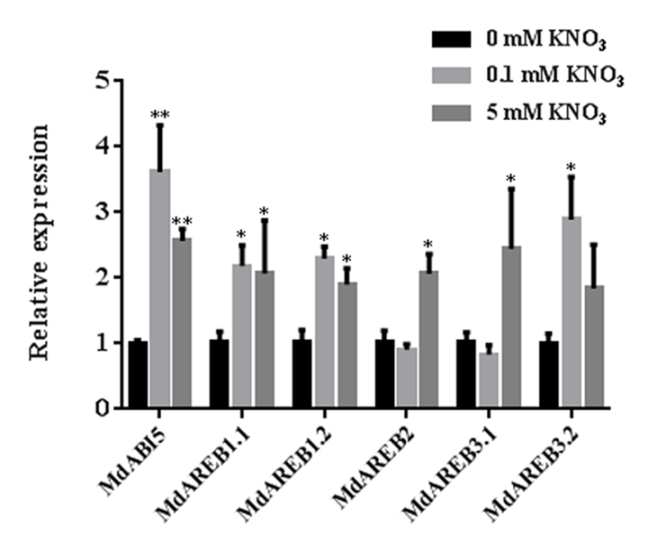


**Fig. S5** **Response of *MdAREBs* to nitrate.** Expression levels of *MdAREBs* in apple seeding roots under different nitrate conditions (0 mM KNO_3_, 0.1 mM KNO_3_ and 5 mM KNO_3_). Data were shown as means ± SE (n =3). Student’s t test (*P < 0.05 and**P < 0.01) was used to analyze statistical significance.

**
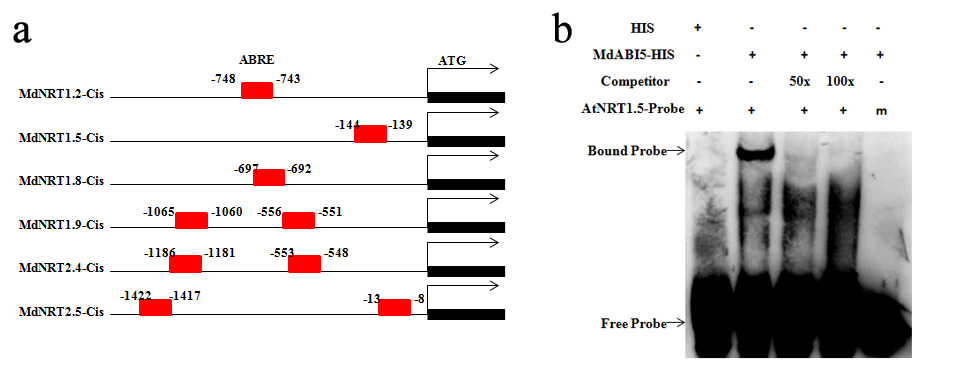
**

**Fig. S6 MdABI5 binds directly to the promoter of *AtNRT1.5.* a** Schematic diagram of the *cis*-element in MdNRT1.2 (MD16G1079100), MdNRT1.5 (MD06G1029400), MdNRT1.8 (MD03G1016400), MdNRT1.9 (MD06G1186500), MdNRT2.4 (MD11G1141700), and MdNRT2.5 (MD13G1096700) promoters. Red boxes indicate the ABRE *cis*-element in the promoter of each gene. **b** EMSA of MdABI5 bound to the *AtNRT1.5* promoter in vitro.


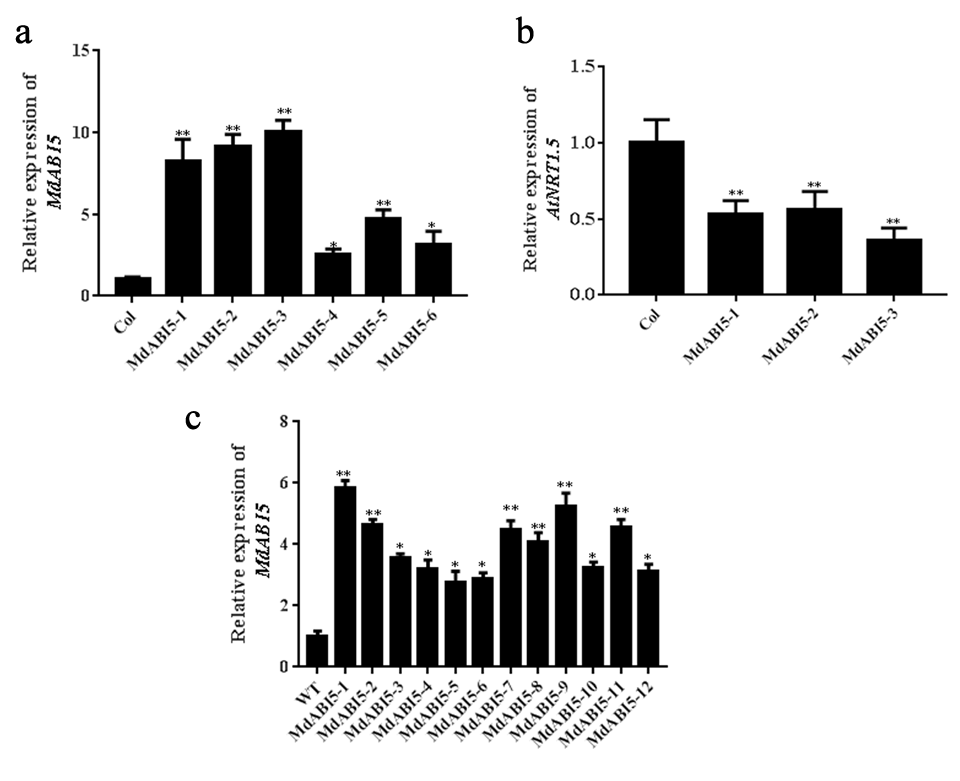


**Fig. S7 Identification of *MdABI5* transgenic plants.** **a** qRT-PCR determination of the *MdABI5* expression level in *MdABI5* transgenic *Arabidopsis*. **b** qRT-PCR determination of the *AtNRT1.5* expression level in *MdABI5* transgenic Arabidopsis. **c** qRT-PCR determination of the *MdABI5* expression level in *MdABI5*-overexpressing apple roots**.** The numbers 1–12 represent different strains.


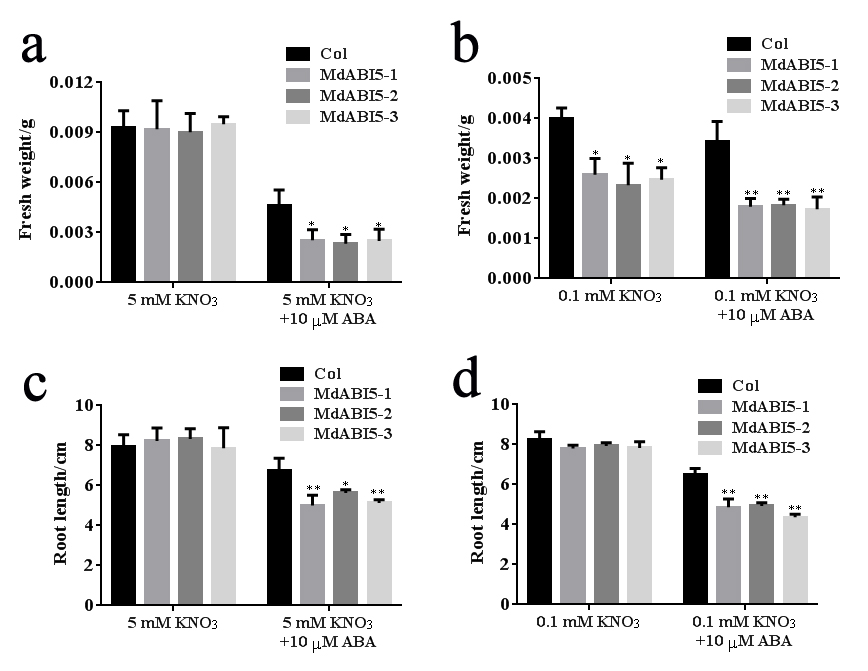


**Fig. S8 Physiological data of *MdABI5* transgenic Arabidopsis treated with nitrate and ABA. a, c** Fresh weight and root length of various plant lines (Col and *MdABI5* overexpressing *Arabidopsis*) and organs (shoot and root) after growth on high nitrogen (5 mM KNO_3_) nutrient solutions containing different concentrations of ABA (0 μM and 10 μM) for 10 d**. b, d** Fresh weight and root length of various plant lines (Col and *MdABI5* overexpressing *Arabidopsis*) and organs (shoot and root) after growth on low nitrogen (0.1 mM KNO_3_) nutrient solutions containing different concentrations of ABA (0 μM and 10 μM) for 10 d.

**
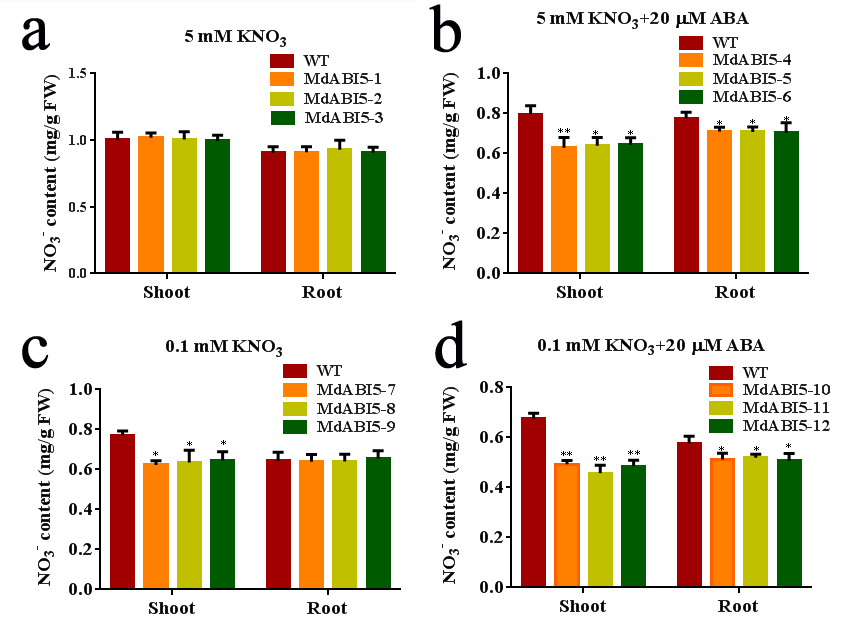
**

**Fig. S9** **a-b** Nitrate contents in various plant lines and organs (shoot and root) after growth on 5 mM KNO_3_ nutrient solutions containing different concentrations of ABA (0 μM and 20 μM) for 50 d. **c-d** Nitrate contents in various plant lines and organs (shoot and root) after growth on 0.1 mM KNO_3_ nutrient solutions containing different concentrations of ABA (0 μM and 20 μM) for 50 d

Table 1. The primers used in this study

| Primer | Sequence (5'to 3') |
| --- | --- |
| MdABI5-F(RT) | GTATCAGAACGGCAACCA |
| MdABI5-R(RT) | GCATATCCATCACCACCA |
| MdNRT1.5-F(RT) | TCGTTGTCGCTGTCCTCG |
| MdNRT1.5-R(RT) | AGCCCTTCCTTGCTGTGC |
| MdNPF4.3-F(RT) | CTCAAGGAAGCATACTCA |
| MdNPF4.3-R(RT)  MdNPF4.4-F(RT) | CAACTGTGGATTAGATGGA  GCAGTCTAAGAAGCTATCGT |
| MdNPF4.4-R(RT) | AAACCCAACATCCATCCCGG |
| MdNPF4.5-F( RT) | CGGACTCGTAGAAACTCG |
| MdNPF4.5-R( RT) | GAAATACCCAAACGCAAT |
| MdNPF5.4-F(RT) | ACATCACTCTGGCATAAC |
| MdNPF5.4-R(RT) | TTCTCATTGGCACTACTG |
| MdNPF6.2-F(RT) | GTTGTGGTGGAAGGAGATT |
| MdNPF6.2-R(RT) | GGTGGACGCTTGTTCTAC |
| MdNRT2.4-F(RT) | AATGGCAGATTCAGAAGGTG |
| MdNRT2.4-R(RT) | CTAAACATGGGAAGGTGTGGT |
| MdAREB1.1-F(RT) | AGCCTAATGGTGCTGGTT |
| MdAREB1.1-R(RT) | GGTTGTTGCTTGGGAAAT |
| MdAREB1.2-F(RT) | GTGTCACCCGTGCCTTAT |
| MdAREB1.2-R(RT) | CCATTTGAGCCTGTTTGTT |
| MdAREB2-F(RT) | GTCGGAAGGGAAGTAAAT |
| MdAREB2-R (RT) | AAAAAGTCCTCCAAAGTCATC |
| MdAREB3.1-F(RT) | ACAGTTGCCACAAGGTCA |
| MdAREB3.1-R(RT) | TCCCAGGTGTCTGCGTAT |
| MdAREB3.2-F(RT) | CTGCAGGCCGAGGAGAATTAAT |
| MdAREB3.2-R(RT) | GATACACACACACACACACACAC |
| MdABI5-FR-F | GGTGAAGGGCTCCTTCTTAAGATGTGCTTCAATTTGATCGGGT |
| MdABI5-FR-R | CAAACCACAACTATGACTCCTCAAGGGTGGGCGCGC |
| MdNRT1.2-F(Chip) | AAGTTTCACCTACTCCCC |
| MdNRT1.2-R(Chip) | :CAAATTTAACAGCAGCCCC |
| MdNRT1.5-F (Chip) | ACTTCAATACTCAGCCAGAC |
| MdNRT1.5-R (Chip) | TTGGACAAACAAAGAGGG |
| MdNRT1.8-F(Chip) | CATTATGCTTAGTGATGA |
| MdNRT1.8-R(Chip) | AAACTAGGTCGTTTCTTC |
| MdNRT1.9-F1(Chip) | ATGGGCTTGTATTTATAG |
| MdNRT1.9-R1(Chip) | ACCAGTTATGTGCTTCTT |
| MdNRT1.9-F2(Chip) | TTCCTCTTATGTGCGGTTAT |
| MdNRT1.9-R2(Chip) | ATCGAGCTTTCATTGTCTTC |
| MdNRT2.4-F1(Chip) | ATGGGGGATTTAGGTTGTAGG |
| MdNRT2.4-R1(Chip) | AGAGTTGACGTGGTAGCC |
| MdNRT2.4-F2 (Chip) | ATGCGGATGATTGCTTGT |
| MdNRT2.4-R2(Chip) | TTAACTCTGCTCGGGACT |
| MdNRT2.5-F1(Chip) | AAAGAATAATAGAGCACGTG |
| MdNRT2.5-R1(Chip) | GTAGAAAGTTTGAAAGGAAT |
| MdNRT2.5-F2(Chip) | CAGGAGTGATTAAGACTTG |
| MdNRT2.5-R2(Chip) | ATTAAGGTACGTGGAGGTT |
| MdNRT1.2-F(EMSA) | GTTTCACCTACTCCCCACACGTTCTATCTCTCCCCC |
| MdNRT1.2-R(EMSA) | GGGGGAGAGATAGAACGTGTGGGGAGTAGGTGAAAC |
| MdNRT1.5-F(EMSA) | ACTCAGCCAGACAAATTGCACGTACACAATATTGGATC |
| MdNRT1.5-R(EMSA) | GATCCAATATTGTGTACGTGCAATTTGTCTGGCTGAGT |
| MdNRT1.5-mF(EMSA) | ACTCAGCCAGACAAATTGACAAACCACAATATTGGATC |
| MdNRT1.5mR(EMSA) | GATCCAATATTGTGGTTTGTCAATTTGTCTGGCTGAGT |
| MdNRT1.8-F(EMSA) | TATCAAAAGGTCCTTTTCACGTGGGAATCAAATAGCAT |
| MdNRT1.8-R(EMSA) | GTCGACTCTAGATCGGATCGGATGAATTG |
| MdNRT1.9-F1(EMSA) | TCGTTTCTGCCAGGTGTTGACACGTGTCCTATTTGATGA |
| MdNRT1.9-R1(EMSA) | TCATCAAATAGGACACGTGTCAACACCTGGCAGAAACGA |
| MdNRT1.9-F2(EMSA) | TTTCGTTGAGTCACACGCTACGTGTAAAATTTATGTAA |
| MdNRT1.9-R2(EMSA) | TTACATAAATTTTACACGTAGCGTGTGACTCAACGAAA |
| MdNRT2.4-F(EMSA) | TTTGCCACGTGGCACAAATTTGGCTACCACGTCAACTC |
| MdNRT2.4-R(EMSA) | GAGTTGACGTGGTAGCCAAATTTGTGCCACGTGGCAAA |
| 18S -F | ACACGGGGAGGTAGTGACAA |
| 18S -R | CCTCCAATGGATCCTCGTTA |
